# Strategic decision making about travel during disease outbreaks: a game theoretical approach

**DOI:** 10.1098/rsif.2018.0515

**Published:** 2018-09-12

**Authors:** Shi Zhao, Chris T. Bauch, Daihai He

**Affiliations:** 1 Department of Applied Mathematics, Hong Kong Polytechnic University, Kowloon, Hong Kong; 2 Department of Applied Mathematics, University of Waterloo, Guelph, Canada

**Keywords:** game theory, travel, infectious disease, decision making, mathematical modelling

## Abstract

Visitors can play an important role in the spread of infections. Here, we incorporate an epidemic model into a game theoretical framework to investigate the effects of travel strategies on infection control. Potential visitors must decide whether to travel to a destination that is at risk of infectious disease outbreaks. We compare the individually optimal (Nash equilibrium) strategy to the group optimal strategy that maximizes the overall population utility. Economic epidemiological models often find that individual and group optimal strategies are very different. By contrast, we find perfect agreement between individual and group optimal strategies across a wide parameter regime. For more limited regimes where disagreement does occur, the disagreement is (i) generally very extreme; (ii) highly sensitive to small changes in infection transmissibility and visitor costs/benefits; and (iii) can manifest either in a higher travel volume for individual optimal than group optimal strategies, or vice versa. The simulations show qualitative agreement with the 2003 severe acute respiratory syndrome (SARS) outbreak in Beijing, China. We conclude that a conflict between individual and group optimal visitor travel strategies during outbreaks may not generally be a problem, although extreme differences could emerge suddenly under certain changes in economic and epidemiological conditions.

## 1. Introduction

Visitors can play an important role in the transmission and spread of infectious diseases. They can serve as susceptible hosts and be infected while staying in one place and then act as mobile sources of case imports to other populations [1–3]. On the one hand, more visitors can lead to substantial benefits for the local economy and businesses. On the other hand, some infectious diseases spread aggressively in major tourism destinations (e.g. Hong Kong, New York, Singapore, Toronto, Beijing), and a large number of visitors can have unexpected impacts on public health [3–5]. For example, severe acute respiratory syndrome (SARS) was introduced to Beijing, China by a few infected visitors in early March 2003, resulting in a large epidemic [6–11]. Other examples where visitors have played a role in regional or international spread include pandemic influenza [12–14], Ebola fever [15] and Middle East respiratory syndrome coronavirus (MERS-CoV) [16]. Enforcing restrictions on incoming visitors could be an efficient way to control local disease outbreaks [7,17–19], but the decision to restrict visitors must be weighed carefully due to the economic and social repercussions.

Game theory attempts to analyse situations where individuals must make decisions in a group environment and where each individual's decision influences the pay-off received by the others in the group [20]. Many interventions (such as vaccination and social distancing) create positive externalities, i.e. benefits to those who did not participate in the intervention, because of herd immunity generated by interruption of transmission. Hence, many previous models have illustrated the discrepancy between the optimal individual strategy that maximizes personal interest, and the strategy that serves the group best by minimizing the overall health burden on the population [21–26]. Although several factors may alter this picture and have been explored in successive work—such as the beneficial effects of social norms and prosocial vaccination [27,28]—these models often illustrate a conflict between group and individual optima across a very broad region of parameter space, covering most epidemiologically and economically relevant regimes [21,22,24,25].

However, this previous research has been mostly concerned with individuals making decisions in a closed population where the disease is already established and is spreading [21–25,29–33], and does not consider multipopulation interactions or the strategic considerations faced by a visitor deciding whether to travel to an affected area during an outbreak. In the context of travel decisions, game theory can be used to answer questions such as whether travelling or not travelling to a location is optimal according to a criterion of self-interest, and the answers it provides can be contrasted with optimal control strategy from the health authority perspective, in terms of maximizing overall population utility.

In this work, we incorporate an epidemic model (based on the classic susceptible–infectious–recovered (SIR) model) into a game theoretical framework to investigate the effects of strategic decisions about travel on local disease control. In contrast with many previous game theoretical analyses of decision making in epidemiological systems in a closed population, for this visitor's game, we find perfect agreement between the individual and group optimal strategies for a range of epidemiologically and economically plausible parameter values. This agreement can be observed in two forms: individual and group optimal strategies both completely reject travelling when the real or perceived disease risk level is sufficiently high, or both strategies allow free travel when the real or perceived disease risk level is sufficiently low. However, disagreement (or conflict) between the individual visitor strategy and the group optimal strategy is observed in two forms: an overload or deficit of visitors compared to the group optimum. In regions where disagreement occurs, the disagreement between the individual optimum (corresponding to a ‘voluntary entrance’ scheme) and the group optimum (corresponding to a ‘restricted entrance’ scheme) is significant. During an outbreak, this conflict is likely to occur at any real or perceived disease risk level. More importantly, in this region, the model outcomes are highly sensitive to small changes in infection transmissibility and visitor costs/benefits. For certain parameter regimes, uncontrolled visitor inflow could result in unexpected large-scale outbreaks when the disease risk level suddenly increases by even a small amount, and the local health authority's travel restrictions could effectively control disease outbreaks when visitor inflow is considered to be ‘overloaded’ during epidemics. Interestingly, the faster the disease risk information is updated, the more likely a discrepancy will occur. Moreover, faster updating of the disease risk information could effectively prevent visitor inflow ‘overload’ and therefore stop an outbreak.

The remaining parts of this work are organized as follows. In the next two sections, we establish a game theoretical framework and an epidemic model including both travelling and local populations, to model the individual decision-making process. In the subsequent sections the results are presented along with a detailed discussion.

## 2. Travelling game

**Figure 1.**
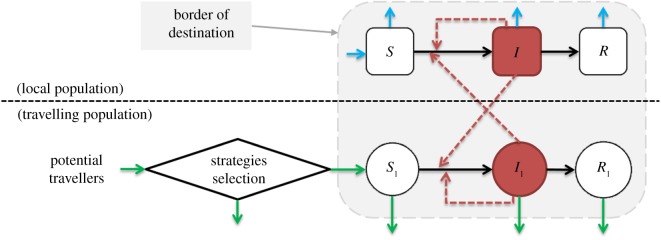
The epidemic model diagram. Black arrows represent infection status transition paths and red dashed arrows represent transmission paths. The light blue arrows represent natural births and deaths, and green arrows represent visitor entry and exit. Square compartments represent local classes, circular compartments represent visitor classes, and the diamond denotes the ‘decision’ process of potential visitors. Red compartments represent infectious classes. The light grey area (surrounded by a grey dashed line) represents ‘inside border’. The horizontal black dashed line separates the total population into ‘local population’ (or local residents) and ‘travelling population’ (as in Path 2.1). (Online version in colour.)

Our game is a population game where players are individuals in a homeland population (the ‘travelling population’) deciding whether or not to travel to an affected destination. These individuals can move through the following states:2.1
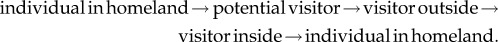
A certain fraction of individuals in a homeland population are designated as potential visitors, who have the economic means and opportunities for travel. A potential visitor may adopt a strategy of travelling to the destination and leaves their homeland, becoming a ‘visitor outside’. Upon arrival at the destination, they become a ‘visitor inside’, and subsequently they become a ‘removed visitor’ and re-join the homeland population, again as a potential visitor. A potential visitor corresponds to *N*_1_ in table 1, a visitor outside corresponds to *ρN*_1_ in the term *f*(*ρ*) in equation (3.1), a visitor inside corresponds to (*S*_1_ + *I*_1_ + *R*_1_) in electronic supplementary material, S3, and an individual in homeland means that a visitor has been removed from the system and re-joins individuals in the homeland. More details of the steps individuals may take in travelling can be found in electronic supplementary material, S1. Figure 1 presents the process of a ‘travelling’ individual joining the epidemic system (i.e. from ‘potential visitor’ to ‘individual in homeland’).

**Table 1.** Summary table of model parameters. The ranges of the parameters are used for the sensitivity analysis. The point values of the disease parameters reflect influenza, and the ranges of the parameters reflect a broad range of other infectious diseases. The values and ranges of the parameters related to travel (i.e. *K*_1_, *r*, *ν*^−1^ and λ^−1^) reflect Hong Kong as the default destination.

parameter	notation	value	range/remark	source(s)
basic reproduction number	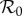	2.5^a^	[1.0, 10.0]	[34–37]
mean duration that visitors are outside border	λ^−1^	3 days	[0.1, 10]	S6.1
ratio: 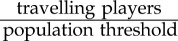	*N*_1_	7.5%	[5.0%, 15.0%]	assumed, S2.2 and S3
ratio: 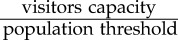	*K*_1_	7.0%	[5.0%, 15.0%]	S6.2
mean infectious period	*γ*^−1^	5 days	[2.0, 10.0]	[38]
mean human lifespan	*μ*^−1^	70 years	fixed	—
mean duration that visitors are inside border	*ν*^−1^	3 days	[0.5, 15.0]	S6.3
relative risk (as in equation (2.5))	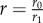	10^−3^	[10^−4^, 10^−2^]	S6.4
probability of travelling	*p*	—	[0.0, 1.0]	equation (2.4)
optimal probability of travelling	p*	—	[0.0, 1.0]	S2.1
proportion of visitors	*ρ*	—	[0.0, 1.0]	equation (2.2)
optimal proportion of visitors	ρ*	—	[0.0, 1.0]	equation (2.6) and S2.2
cost of all game players	*Υ*	—	—	S2.2
difference between group and individual optima	Δ*ρ*	ρ* − p*	[−1.0, 1.0]	equation (4.1)
probability that a disease outbreak occurs	*α*	0.01^b^	[0.001, 0.02]	assumed

^a^One can determine the function 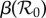 explicitly from equation (3.2), and 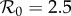 is also applicable to the 2003 SARS epidemic according to [6,7,9–11,39].

^b^*α* = 1.0 during epidemics.

For simplicity, we suppose that every individual receives the same information and picks strategies in the same way (i.e. with equivalent preferences and equivalent pay-off for the same strategy). An individual can decide whether to travel (i.e. the ‘travelling’ strategy) or not to travel (i.e. the ‘non-travelling’ strategy) to their destination. We use *r*_1_ to denote the perceived cost (negative pay-off) of morbidity and/or mortality risk (i.e. the risk of disease, or as a term of ‘health cost’) from infection. Similarly, we use *r*_0_ to denote the perceived cost of the risk of utility loss for adopting the ‘non-travelling’ strategy, since those individuals lose economic or social opportunities. Therefore, we write the pay-off for an individual following the travelling strategy as2.2
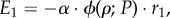
where *α* represents the probability that an epidemic occurs at the destination during a traveller's visit (or, *α* = 1 for an ongoing epidemic that the traveller knows about before departure), *ϕ*(*ρ*; *P*) is the probability that a visitor is infected during the trip (to the epidemic destination) given that the pre-existing immunity level in the destination population is *P*, and *ρ* is the overall proportion of potential visitors who adopted the ‘travel’ strategy.

To assess the risk of a visitor being infected during the trip, we need to know the basic reproduction number of the disease, 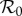, i.e. the expected number of secondary cases generated by a typical primary case during his/her infectious period in an otherwise susceptible population. In the case of 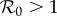, we have *ϕ*(*ρ*; *P*) = 0 if 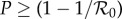 (see electronic supplementary material, S2.1). This is called perfect herd immunity, i.e. an outbreak cannot occur when the population immunity level is greater than 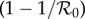 [40,41]. We denote the pay-off of an individual following the non-travelling strategy as2.3
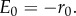
Since this is a population game, we also define a mixed strategy where players follow the travelling strategy with a probability *p* and follow the non-travelling strategy with a probability (1 − *p*). The pay-off function is then2.4
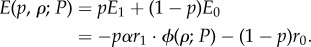
The game remains unchanged if we scale the pay-off function by a constant; thus, we eliminate one parameter in equation (2.4) by leaving only the relative risk, *r* = *r*_0_/*r*_1_. Normally, we have 0 < *r*_0_ ≪ *r*_1_ since the pay-off of utility loss, *r*_0_ in equation (2.3), should be less than that of health loss, *r*_1_ in equation (2.2), if the disease is severe or potentially deadly. Hence we assume 0 < *r* ≪ 1 in general. Furthermore, we have2.5
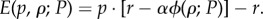
For convenience, we denote *ϕ*(*ρ*; *P*) as *ϕ*(*ρ*) and *E*(*p*, *ρ*; *P*) as *E*(*p*, *ρ*) and fix *P* in the rest of this work. We can show that the individual equilibrium (p*) of the game exists, is the unique Nash equilibrium, and is stably convergent (see electronic supplementary material, S2.1).

We formulate the (scaled) costs of all potential visitors (game players) as2.6
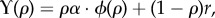
where all terms have the same meaning as in equation (2.5). More details are provided in electronic supplementary material, S2.2. We also define the group (Pareto) optimum ρ* as the value of *ρ* for which the population average cost function *Υ*(*ρ*) of all potential visitors (i.e. all game players) is minimized.

## 3. Epidemic model

### 3.1. Formulation of epidemic model

To specify the infection probability *ϕ*(*ρ*), we adopt the standard SIR model. Individuals of the destination population (excluding visitors) are categorized as susceptible to the disease (*S*, those who may be infected), infectious (*I*, those capable of transmitting disease) or removed (*R*, those who are either recovered and immunized or died). Similarly, visitors are also categorized as susceptible (*S*_1_), infectious (*I*_1_) or removed (*R*_1_). We use *S*, *I* and *R* (*S*_1_, *I*_1_ and *R*_1_) to denote the proportions of susceptible, infectious and recovered individuals in the destination (visitor) populations, respectively. This patchy population structure was proposed previously in [1,2,42,43]. Before taking the trip, visitors are assumed to be totally susceptible. We illustrate this ‘local-and-travelling population’ interactive epidemic system in figure 1. We further assume that the susceptible visitors follow a logistic growth mechanism.

— The visitor population capacity (e.g. the number of beds in hotels) of one place is finite and assumed to be a constant.
— Low (/high) volume of visitors will increase (/decrease) the recruitment effort of travellers for a business trip and decrease (/increase) the expense for a recreation trip.

Thus, logistic growth is a reasonable choice. After eliminating *R*′ and *R*′_1_ (see electronic supplementary material, S3 for details), we formulate the epidemic model as3.1
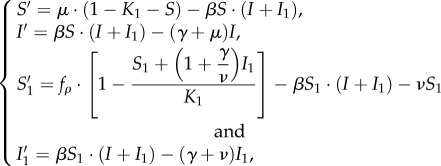
where *f*_*ρ*_ = *f*(*ρ*) = *ρ*λ*N*_1_ represents the rate of incoming visitors, *K*_1_ is the maximum visitor capacity that the destination is willing (or able) to accept, *N*_1_ is the number of all players (i.e. all potential visitors), and players who adopt the ‘travel’ strategy, travel from the homeland to the destination at a rate 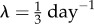 (see electronic supplementary material, S6.1). We express both *K*_1_ and *N*_1_ in units of proportion of the population threshold (destination population plus the maximum visitor capacity) and we fix *N*_1_. We assume that all trips are 3 days long, hence visitors return at rate 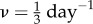 (see electronic supplementary material, S6.3). We summarize all model parameters in table 1.

The contact term *β* is a function of 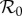. Using the next generation matrix method [44], we derive the basic reproduction number of our epidemic model as3.2
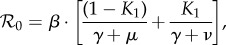
thus, 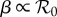 when the values of the other parameters are fixed.

### 3.2. Model equilibria

We denote the disease-free equilibrium (DFE) as
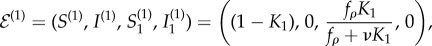
where *I* = *I*_1_ = 0 and *S*^(1)^_1_ < *K*_1_. The DFE (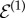) is globally stable when 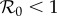, whereas it is unstable when 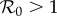. When 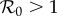, there is an endemic, i.e. the visitor-absent endemic equilibrium,
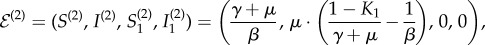
where *S*_1_ = *I*_1_ = 0. Specifically, *S*^(1)^ = (*γ* + *μ*)/*β* is the reciprocal of 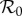 of the standard SIR model [41]. 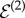 can be realized when *f*_*ρ*_ in *S*′_1_ (see equation (3.1)) becomes 0 and it is locally stable. When 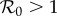, there also exists another endemic equilibrium corresponding to a mixed state of local and visitor infections (i.e. infected visitors), denoted as 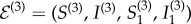. The solution of 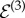 can be obtained explicitly by taking the non-negative root of [*S*′, *I*′, *S*′_1_, *I*′_1_]^T^ = **0** (**0** represents the zero vector) with both *I* ≠ 0 and *I*_1_ ≠ 0.

### 3.3. Probability of visitors becoming infected

Given the model in equation (3.1) and the assumption that all individuals in a compartment leave it at the same rate regardless of how long they have been there, we may take the probability of a visitor becoming infected during the trip to be equal to the ratio of the rate at which susceptible visitors (*S*_1_) are infected to the rate at which susceptible visitors (*S*_1_) leave the destination [22],3.3
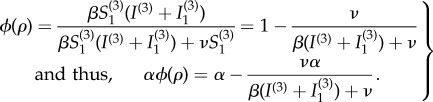
We present the numerical results of the relationship between *ϕ*(*ρ*) and *ρ* in electronic supplementary material, S2.1. Given the relationship between *β* and 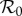, one may derive the relationship between 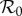 and *ϕ*(*ρ*) explicitly.

## 4. Results and discussion

### 4.1. Individual equilibrium and travelling optimum

**Figure 2.**
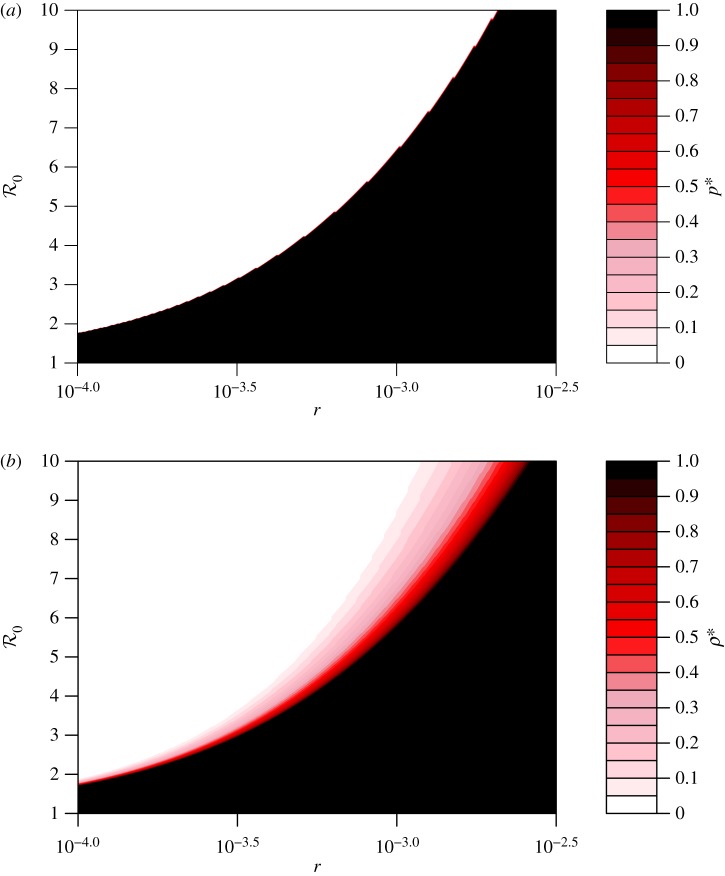
Individual and population optima as functions of the basic reproduction number 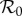 and the relative risk *r* during an epidemic (*α* = 1). (*a*) The Nash equilibrium proportion of travellers p*; (*b*) the group optimal proportion of travellers ρ*, with colour codes to indicate magnitude. The range of 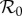 and the values of the other parameters are listed in table 1. (Online version in colour.)

We first explore how the predicted travel strategies depend on the basic reproduction number (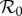) and the relative risk (*r*). Many factors, including seasonal (climatic) factors and the evolution of viruses, could affect 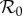. Additionally, media coverage of the risk and relevant educational programmes [45–50] could influence visitors' perception of the risk, thus changing *r*_1_ and *r* (equation (2.5)). During an ongoing epidemic (*α* = 1), we find that both *r* and 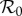 significantly influence the individual equilibrium p* and the group optimum ρ* (figure 2). (The values of the other parameters are fixed and listed in table 1, and small variations in their values do not dramatically change the trends of these relationships.) We observe that both the individual and population optima have the same qualitative relationship with 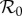 and *r*: both optima are monotonically decreasing functions of 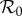 and monotonically increasing functions of *r*. This behaviour is expected, since an increasing transmissibility should reduce both the individual incentive to travel and the group optimal rate of travelling, while a decline in the relative risk of travelling should encourage travel, both individually and as a group. More surprisingly, the sudden transition of the individual optimum from 0 to 1 (as shown in figure 2*a*) is steeper than that of the population optimum (as shown in figure 2*b*).

**Figure 3.**
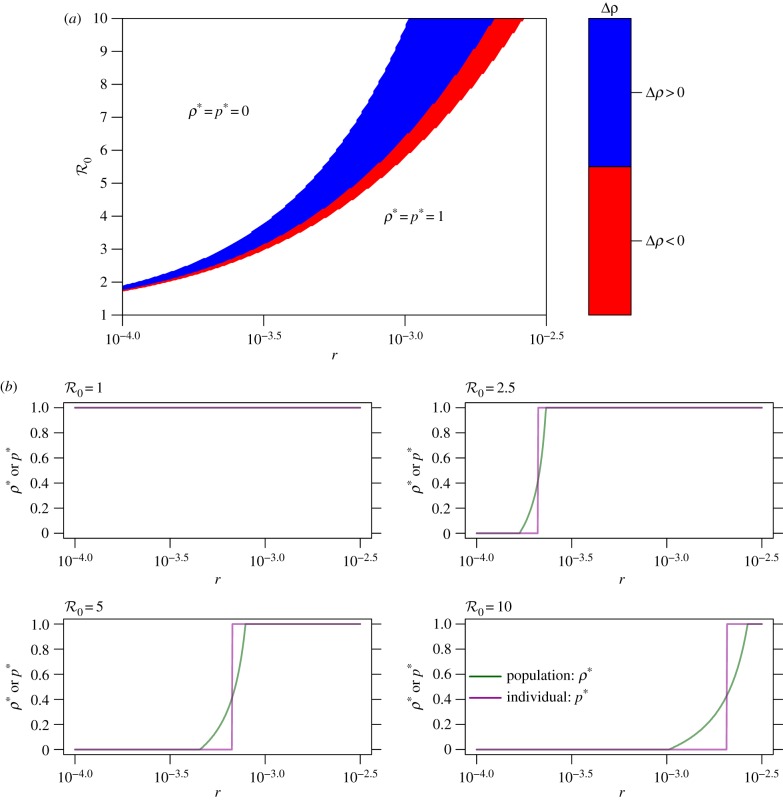
Discrepancy between individual and population optima as a function of the basic reproduction number 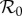 and relative risk *r*, during an epidemic (i.e. *α* = 1). (*a*) The relationship among *r* (equation (2.5)),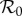 and Δ*ρ* (equation (4.1)); and (*b*) the relationship between *r* and Δ*ρ* for 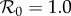, 2.5, 5.0, 10.0. In (*a*), the colour code quantifies Δ*ρ*. The white area represents Δ*ρ* = 0 under the two cases that ρ* = p* = 0 or 1. In (b), ρ* is in green and p* is in purple. In (*a*,*b*), the range of 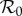 and the values of the other parameters are listed in table 1. (Online version in colour.)

To further explore the relationship between the individual and group optimum, we study their difference:4.1
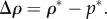
More details are given in electronic supplementary material, S2. A plot of Δ*ρ* versus the population optimum ρ* and the individual equilibrium p* during an ongoing epidemic (*α* = 1) show that they agree perfectly for most of the parameter space (figure 3). For most of the parameter region, ρ* = p* = 0 or 1 (i.e. the white area in figure 3). These two situations can occur when both the disease risk (reflected by 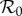) and perceived risk are (i) either considerably high, i.e. ρ* = p* = 0, in which case no one intends to travel and complete border entrance restrictions are implemented, or (ii) considerably low, i.e. ρ* = p* = 1, in which case all individuals intend to travel and border entrance is completely unrestricted. Variations in the values of the other parameters do not change the trends of these relationships (table 1).

However, despite the broad agreement across the parameter plane, the region where ρ* and p* are discrepant reveals interesting findings. During an epidemic, most locations are expected to receive fewer visitors (with limited visitor entrance) than usual when there is no epidemic. But the model predicts parameter regimes where the group optimal solution requires a higher volume of travel than what is individually optimal: in the blue region of the parameter plane, Δ*ρ* > 0, meaning p* < ρ* (figure 3*a*). In this regime, the health authority would wish to encourage more travel than actually occurs. However, if either the disease risk 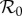 or the perceived pay-off of disease risk *r*_1_ decline even slightly (for instance, due to seasonal factors and/or changing media coverage) the situation is reversed, and the discrepancy in interests Δ*ρ* could change from Δ*ρ* > 0 to Δ*ρ* < 0 (red region in figure 3*a*). When Δ*ρ* < 0, a health authority restriction on visitors is desired and only ρ*/p* of the visitors should be allowed to enter in order to achieve the population optimum ρ*. In summary, figure 3 shows a surprising contrast to many game theoretical models comparing individual and group optimal outcomes: in large parts of the parameter space, there is no discrepancy. However, when a discrepancy does emerge, it can emerge very quickly with small changes in parameter values, and moreover, the individual optimal travel rate could exceed the group optimal rate, or vice versa.

### 4.2. Example of the 2003 severe acute respiratory syndrome outbreak in Beijing

**Figure 4.**
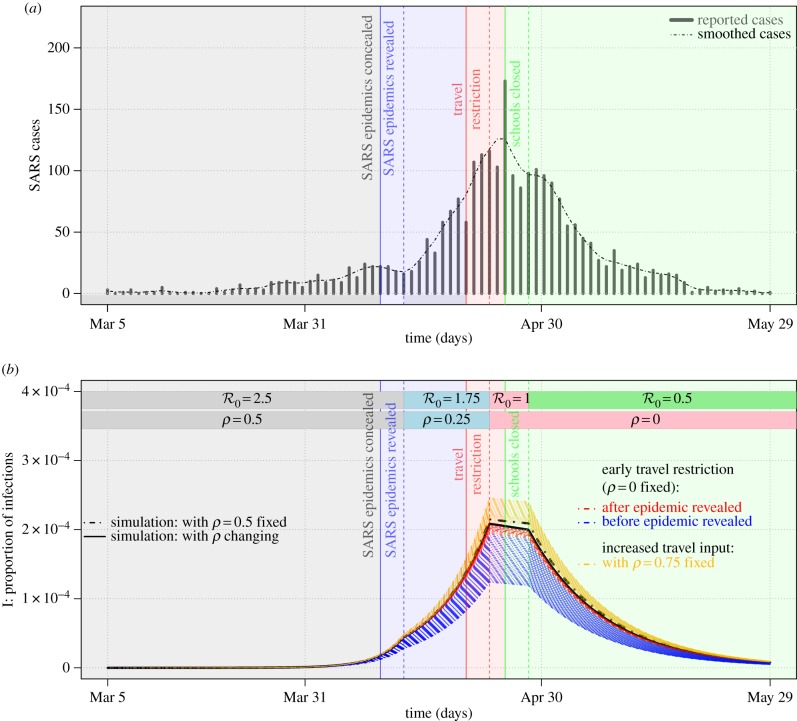
The 2003 SARS outbreak in Beijing, China. (*a*) The reported cases during the 2003 SARS outbreak in Beijing, China (adapted from Pang *et al*. [11]) and (*b*) the numerical results of the epidemic model (see equations (3.1)). In (*a*,*b*) the vertical lines represent the starting points of events, and the vertical dashed lines represent the time points with a lag of 3 days. In (*a*) the SARS epidemic and government intervention are given on a timeline from 5 March to 29 May 2003. The black dashed line is the time series smoothed by using the *LOESS* function (R v. 3.4.3 ). In (*b*) the initial states are set as [*S*(0), *I*(0), *S*_1_(0), *I*_1_(0)] = [(1 − *K*_1_), 0, (*K*_1_ − 1 × 10^−8^), 1 × 10^−8^], with 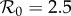, *N*_1_ = 15% and *ρ* = 0.5 (see grey parts of the bars on the top). The blue and red dashed lines are the simulations under ‘what if’ scenarios in which travel restriction policies were implemented earlier. The black and gold dashed lines are under ‘what if’ scenarios in which travel restriction (or reduction) failed and travel input suddenly increased, respectively. The values of the other parameters are assumed to be the same as those in table 1, and the changes in parameters are marked at the top of the panel. Note that the timelines are the same in (*a*,*b*). (Online version in colour.)

The epidemic patterns predicted by our model under a manipulation of the group optimal strategy *ρ* are qualitatively similar to the epidemic curve during the 2003 SARS outbreak in Beijing, China, resulting from the timing of certain travel-related events during the outbreak. Figure 4*a* (adapted from Pang *et al*. [11]) shows weekly reported cases in Beijing during the outbreak. Data are available from the electronic supplementary material. The time point when knowledge of the epidemic was first made public, e.g. *SARS made reportable (Apr 10)* in fig. 1 of Pang *et al*. [11], refers to the date of news press.^1^ The time point of the official start of restrictions on travel refers to the events *outbreak announced publicly by government (Apr 20)* and *fever checks at airports begin (Apr 22)* in fig. 1 of Pang *et al*. [11]. We note that these two events resulted in almost no one travelling to Beijing, i.e. *ρ* = 0, until the end of the SARS epidemic.^2^

We also note that, although the Beijing SARS outbreak was initially sparked by travellers, the proportion of cases in Beijing caused by travellers over the entire outbreak is thought to be small, especially after fever screening began [51]. Also, the United States Centers for Disease Control suggests that travellers to SARS-affected destinations take precautions to avoid infection, suggesting a non-trivial infection risk for travellers.^3^ The latter two features of the Beijing SARS outbreak are consistent with our model assumptions.

Figure 4*b* shows a model-simulated epidemic curve that largely matches the observed epidemic curve. To generate this curve we focus on changes in 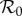 (disease transmissibility) and *ρ* (proportion of players adopting the ‘travel’ strategy). We decrease *ρ* from 0.5 to 0.25 at the time indicated by the blue dashed vertical line in figure 4*b*. This decrease is associated with the start of public awareness of the SARS risk in Beijing after it was revealed to the public (see endnote 1). Similarly, the decrease in 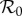 from 2.5 to 1.75 as also indicated by the blue dashed vertical line would correspond to an accompanying reduction of the effective contact rate due to the onset of public awareness of SARS. (The effective contact rate is defined as the product of the contact rate and transmission probability per contact. It is believed, and is modelled, to be negatively, or at least non-positively, related to reported disease incidence [42,46–48,52].) The time lag, i.e. the gap between the pairs of vertical solid and dashed lines of the same colour in figure 4, is fixed at 3 days due to the mixed effects of the incubation period (or the latent period) of SARS infection and the delay of human reaction to the outbreak. The model simulation largely captures the observed SARS epidemic between March and May 2003, as shown in figure 4*a*,*b* and fig. 8 of [53].

The model-predicted outcome of an earlier implementation of travel restrictions (see blue and red dashed lines in figure 4*b*) is obtained by fixing the combinations of 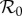 and *N*_1_, and setting *ρ* = 0 (i.e. nobody is able or willing to enter due either to travel restrictions or cautious behaviour due to SARS risk). We found that the earlier the travel restrictions are implemented, the more effectively the disease outbreak level is reduced. By contrast, an uncontrolled and sudden increase in the proportion of visitors (e.g. increasing *ρ* from 0.5 to 0.75) could yield a larger outbreak, as indicated by the gold dashed lines in figure 4*b*.

We note that our objective in figure 4 is to convey how the model framework applies during an unfolding epidemic where travel restrictions are put in place partway through the epidemic. Hence, although the starting value of 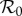 is epidemiologically plausible for SARS [54,55], the parameters were chosen for convenience rather than being fitted systematically. However, slight changes in the parameter values away from this parameter regime do not change the outcomes. Also, additional numerical results for wider parameter variations in electronic supplementary material, S4 show the range of possible dynamics exhibited by the model.

### 4.3. Additional sensitivity analysis

The sensitivity analysis of the baseline model (electronic supplementary material, S5) shows that the results are most sensitive to the relative risk (*r*), basic reproduction number (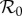) and the rate at which individuals leave the destination (*ν*). More detailed discussion of the influence of these model parameters on model predictions is given in electronic supplementary material, S7.

In the baseline model, for simplicity, we assume that visitors do not bring infection back to their home country. To amend this shortcoming, we introduce an additional probabilistic case importation risk level into an extended model (see parameters in table 1). Under this extension, our main results are unchanged (see electronic supplementary material, S9 for a detailed discussion). We also included pre-existing immunity among visitors in an extended model, and also found that our main results were unchanged. A detailed discussion can be found in electronic supplementary material, S8.

### 4.4. Model limitations and future research

In this subsection, we discuss possible model extensions and some limitations. In the baseline model, we assume individuals have accurate knowledge of the real basic reproduction number 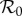. However, an imbalance between the perceived and actual 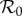 could exist [56–58]. We denote 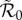 as the perceived 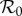. We expect the perceived 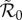 to correlate positively with the actual 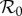. Thus, we assume 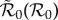 is a non-decreasing function of 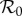. Given the perceived disease risk 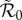, the pay-off of the disease risk 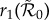, i.e. *r*_1_ as a function of 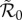 given in equation (2.2), is a non-decreasing function of 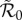 and a non-decreasing function of 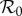. One of the simplest forms of 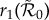 is 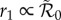 with a positive scalar. Future research should explore the impact of such a difference between 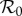 and 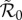.

In addition, travelling players may not always be informed about outbreak events in a timely manner. Thus, a time delay between 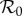 and 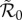 could exist. We denote 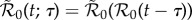, where *τ* ≥ 0 is the time lag between the occurrence of infection risk and the perception of infection risk. If we set *τ* = 0 for all *t* by assuming humans receive accurate knowledge of a risk when it emerges, we have 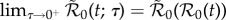. In this work, we consider a limiting case of *τ* = 0. In reality, this assumption can be relaxed, and a reasonable estimate can be used. The value of *τ* depends on the impacts of the risk and the efficiency of the media and relevant programmes (e.g. news press coverage [22,42,46,48], education programmes [22,50,59], communication effectiveness in social networks [49,50,60–63] and pre-existing public health awareness [14,49,61]).

In this work, we assumed the same information availability and the same strategic response for the entire visitor population (see equations (2.2) and (2.3)). However, different groups of people could have different risk perceptions or risk preferences, hence the pay-offs could differ between individuals. This has been demonstrated in previous game theoretical models to lead to different equilibria and optima regarding the human response to epidemics [26,64]. Consider the situation where *E*_1_ = *E*_0_ (see equations (2.2) and (2.3)). In this case, some individuals may prefer the travelling strategy (i.e. risk-seeking preference), while others may prefer the non-travelling strategy (i.e. risk-averse preference).

Future models including a heterogeneous population could improve the realism of the model and help test the robustness of our predictions. One way this could be done is by allowing the disease natural history and economic parameters to vary between individuals (as noted in the foregoing paragraph), to reflect varying health conditions and socio-economic status. Another way to account for heterogeneity at a larger scale is to allow for a patchy environment [1] where different sub-populations are subject to different conditions. Under such circumstances, we expect that the boundaries in figure 2 would probably become less sharp, although it is not clear *a priori* how large the effect would be. We expect that most forms of heterogeneity would not change our finding that the individual and group optima tend to agree in this kind of game theoretical framework, although the regime shifts implied by figure 2 would probably be less dramatic if heterogeneity were included.

## 5. Conclusion

Many game theoretical studies of closed socio-epidemiological systems find a significant discrepancy between individual and group (Pareto) optima in a broad range of economic and epidemiological parameters. In this work, we studied an open socio-ecological system in which visitors decide whether to travel to a location with an ongoing outbreak. Surprisingly, we found perfect agreement between the individual and group optimal strategies for broad ranges of parameter values. When a disagreement between the individual and group optimal strategies occurs, the discrepancy was very large and highly sensitive to small changes in disease transmissibility and visitor costs/benefits. For instance, if disease transmissibility increases by even a small amount, the uncontrolled incoming visitors are capable of causing an unexpected outbreak. This suggests that a discrepancy between the individual and group optima could emerge suddenly in real-world settings, provided that slight changes in economic and epidemiological factors (parameters) occur. However, timely implementation of travel restrictions by health authorities may effectively prevent large-scale outbreaks.

## Supplementary Material

Supplementary Information

## Supplementary Material

Data

## Endnotes

1 News press of the reveal of the 2003 SARS epidemic in Beijing, China: http://www.lifeweek.com.cn/2003/0729/5582.shtml (in Chinese) and http://news.eastday.com/epublish/gb/paper148/20030530/class014800003/hwz953568.htm.

2 The news press of National Tourism Administration (see (in Chinese) http://www.people.com.cn/GB/jingji/1038/1970533.html) and WHO travel advice (see http://www.who.int/csr/sars/archive/2003_04_23/en/)

3 US Centers for Disease Control. Guidance for persons traveling to areas where SARS cases have been reported. See https://www.cdc.gov/sars/travel/advice.html.

## Data accessibility

The 2003 SARS cases time series in Beijing are obtained from Pang *et al*. [11] and are available from the electronic supplementary material.
