# Supplementary material for: Strategic decision making about travel during disease outbreaks: a game theoretical approach
Source: J R Soc Interface. 2018 Sep 12;15(146):20180515. doi: 10.1098/rsif.2018.0515 (PMC6170783; doi:10.1098/rsif.2018.0515)
Supplement: Supplementary Information [file rsif20180515supp1.pdf]

Supplementary Information

Shi Zhao[1], Chris T. Bauch[2,*] & Daihai He[1,*]

1 Department of Applied Mathematics, Hong Kong Polytechnic University, Hong Kong

2 Department of Applied Mathematics, University of Waterloo, Canada

* Correspondence to: daihai.he@polyu.edu.hk (D.H.) and cbauch@uwaterloo.ca (C.T.B.)

August 20, 2018

Contents

S1 More Explanation of the Travelling Procedure **2**

S2 Individual Equilibrium and Group Optimum **2**
S2.1 Individual Equilibrium 2
S2.2 Group Optimum 4

S3 Simplification of the Epidemic Model: Elimination of R and R_1 **5**

S4 Some Numerical Examples **6**

S5 Sensitivity Analysis of Payoffs **6**

S6 Interpretation and Value of Some Model Parameters **7**
S6.1 Rate of visitors moving from outside status to inside status λ 7
S6.2 Visitor capacity at destination K_1 8
S6.3 Rate of visitors leaving destination ν 8
S6.4 Relative risk r 9

S7 Further Discussion of Model Parameters **9**

S8 Pre-existing immunity among visitors **11**

S9 Risk of visitors bringing the disease back to their home country **12**

S1 More Explanation of the Travelling Procedure

In this work, the game players are potential visitors who have a plan to visit a travel destination. Potential visitors will end up in two categories: those who take the trip and those who cancel the trip (see path (1) in main text). Any individual of the home country of visitors may become a potential visitor at any time. The number of potential visitors (or N_1 as the ratio in the model, see Table 1 in main text) is mainly dependent on the travelling pattern (or seasonality) of the destination. A potential visitor may decide to travel (i.e., become a "visitor outside") according to his/her knowledge on the disease risk at the very moment the decision being made. Thus, there are three cases regarding to the different travelling decisions.

- A potential visitor decides to travel and successfully completes the trip. Since the trip is short (three days), we assume that the visitor does not change his/her travel decision. Finally, s/he returns to his/her home population after the trip.
- A potential visitor decides to travel but fails to complete it due to travel restriction at the destination. In this case, the visitor returns to his/her home population.
- A potential visitor voluntarily cancels the trip and stays at his/her home population.

Therefore, in any case, the decision making process of the proposed travelling game follows the sequential game scheme (i.e., the decision is "renewable" for every participant in this game). We note that local "travel restriction" only has its effects on these potential visitors who decide to travel; and the potential visitors is mainly influenced by the travelling pattern to the destination.

S2 Individual Equilibrium and Group Optimum

S2.1 Individual Equilibrium

We assume that a proportion ε $(0 < \varepsilon < 1)$ of potential visitors will take the trip with a probability p (i.e., playing p strategy) and the rest of potential visitors $(1-\varepsilon)$ will take the trip with probability q, where $q \neq p$. Then, the overall proportion of visitors $(\bar{\rho})$ who will take the trip among all game players is

$$\bar{\rho} = \varepsilon p + (1-\varepsilon)q. \tag{S1}$$

Therefore, the payoff to individuals playing p-strategy and q-strategy are $E(p, \bar{\rho})$ and $E(q, \bar{\rho})$, respectively. The payoff gain (or loss if negative) of an individual playing p strategy against q strategy is the difference of two payoff functions,

$$\Delta E = E(p, \bar{\rho}) - E(q, \bar{\rho}) = (p-q)\left[r - \alpha\phi(\bar{\rho})\right]. \tag{S2}$$

where the parameters have the same meaning as in the main text.

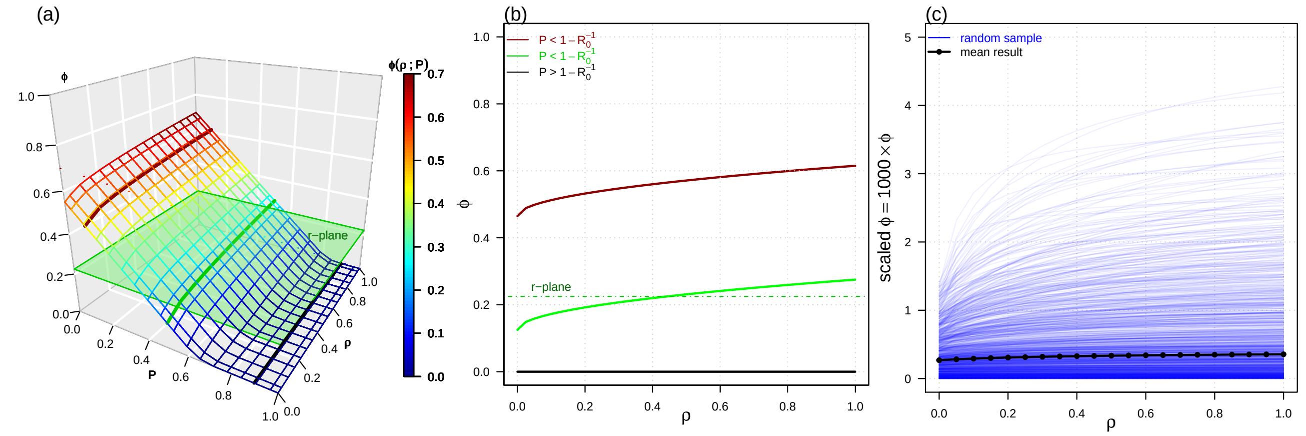

Figure S1: Schematic diagram of Nash equilibria under three "existence" situations (panel (a) and (b)) and numerical results of the relation between $\phi(\rho)$ and ρ (panel (c)). In order to have a clear demonstration of three kinds of Nash equilibria, panel **(a)** and **(b)** show the trends of $\phi(\rho; P)$ against ρ and P. Panel **(c)** shows the relation between scaled $\phi(\rho)$ and ρ. The scaled $\phi(\rho) = 1000 \times \phi(\rho)$. In panel **(c)**, the transparently blue line are from 1,000 random samples with parameter sets, and the black dotted line is the result with fixed parameter values. The parameters' values and ranges can be found in Table 1.

Existence of Nash Equilibria The probability of a visitor becomes infected during the trip $(0 < \phi(\rho) < 1)$ must increase strictly (which is in line with [**?**], as explained in Epidemic Model section) when a proportion ρ of game players choose the travelling strategy (see Fig. S1). Hence, when P is fixed, the minimum of $\phi(\rho)$ occurs at $\rho = 0$ and the maximum of $\phi(\rho)$ occurs at $\rho = 1$. Here, we show the existence of the unique Nash equilibria by achieving $\Delta E > 0$ in Eqn. (S2) under three situations.

- If $\alpha \cdot \min\{\phi(\rho)\} = \alpha\phi(\rho = 0) \geqslant r$, $\alpha\phi(\rho) > r$ for all $0 < \rho < 1$, so for any $0 < \varepsilon < 1$ of Eqn. (S1), $\Delta E > 0$ for any $q \neq p$ if and only if $p = 0$ (such that $p - q < 0$ for all $0 < q < 1$), thus, $p^* = 0$ is the unique Nash equilibrium.

- If $\alpha \cdot \max\{\phi(\rho)\} = \alpha\phi(\rho = 1) \leqslant r$, $\alpha\phi(\rho) < r$ for all $0 < \rho < 1$, so for any $0 < \varepsilon < 1$ of Eqn. (S1), $\Delta E > 0$ for any $q \neq p$ if and only if $p = 1$ (such that $p - q > 0$ for all $0 < q < 1$), thus, $p^* = 1$ is the unique Nash equilibrium.

- If $\alpha \cdot \max\{\phi(\rho)\} = \alpha\phi(\rho = 1) > r > \alpha\phi(\rho = 0) = \alpha \cdot \min\{\phi(\rho)\}$, there exist one and only one p^* such that $\alpha\phi(\rho = p^*) = r$. For all $q < p$, we have $\bar{\rho} < p$ (according to Eqn. (S1)) for any $0 < \varepsilon < 1$ and, similarly, for all $q > p$, we have $\bar{\rho} > p$ for any $0 < \varepsilon < 1$. Hence, for $\alpha\phi(\rho = 1) > r > \alpha\phi(\rho = 0)$, we always have $\Delta E > 0$ for all $q \neq p$ if and only if $p = p^*$, so p^* is the unique Nash equilibrium such that $\alpha\phi(p^*) = r$.

These different situations of the relationship between $\alpha\phi(\rho)$ and r are due to different values of the pre-existing immunity level (i.e., P, Fig. S1) and different values of parameters (Table 1).

Convergent Stability Follow the previous work [3], let p be closer to p^* than q (i.e., the unique Nash equilibrium of Eqn. (S2)), which means $q < p \leqslant p^*$ or $q > p \geqslant p^*$ (note that p is not

necessarily equal to p^*). Given $\phi(\rho)$ increases with respect to ρ, if $q < p \leqslant p^*$, $(r - \alpha\phi(\bar{\rho})) > 0$ for all ε in Eqn. (S1), we have $\Delta E > 0$. Similarly, we can also have $\Delta E > 0$ if $q > p \geqslant p^*$ as desired. Therefore, the Nash equilibria in all of the three scenarios are convergently stable.

S2.2 Group Optimum

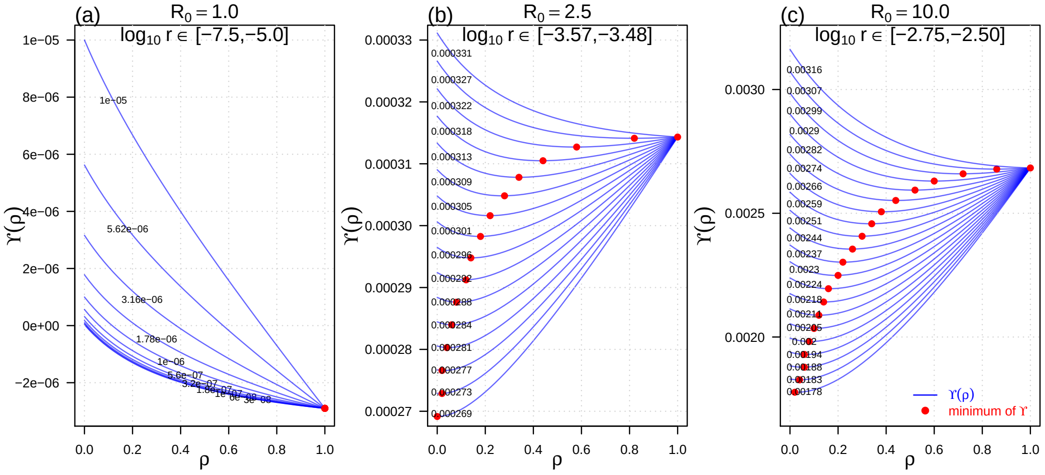

Figure S2: The optimal proportion of travelling-players becoming visitors (i.e., ρ^* corresponding to Eqn. (S4)) during epidemic (i.e., $\alpha = 1$). Panel (a)-(c) corresponds to $\mathcal{R}_0 = 1.0, 2.5$ and 10.0 respectively. Blue lines are $\Upsilon(\rho)$ in Eqn. (S4) with respect to different values of r and red dots are the minima (when $\rho = \rho^*$) of $\Upsilon(\rho)$, of which $\rho \in [0, 1]$. The values of r are shown on each blue line. The range of $\mathcal{R}_0$ and values of other parameters are on Table 1.

For all potential visitors, we aim to minimize the overall cost (negative payoff) of all players, which also appears to be the goal of governmental control. We further ignore the possibility that infected visitors bring the disease back to their home population. We can express the expected cost in term of ρ (i.e., the overall proportion of all players who choose to travel),

$$\Upsilon(\rho) = N_1 \cdot [\rho\alpha \cdot \phi(\rho) \cdot r_1 + (1 - \rho)r_0] \,. \tag{S3}$$

Here, N_1 is the ratio of total number of players to the total local population capacity (i.e., sum of local visitors capacity and number of population). The other terms have the same meaning as in Eqn. (S2). We further scale $\Upsilon(\rho)$ by eliminating N_1 (because N_1 can be fixed as a constant) and one risk term (replacing r_0 and r_1 by $r = \frac{r_0}{r_1}$ [2, 6]. Thus, the (scaled) cost of potential visitors is

$$\Upsilon(\rho) = \rho\alpha \cdot \phi(\rho) + (1 - \rho)r, \tag{S4}$$

where all terms have the same meaning as in Eqn. (S2). The optimal travelling proportion is the optimal ratio of successful visitors over all game players, which is denoted by ρ^*. ρ^* can be obtained by minimizing $\Upsilon(\rho)$ (see Fig S2 as numerical examples).

S3 Simplification of the Epidemic Model: Elimination of R and R_1

Based on the framework of the standard SIR compartmental model (see "Epidemic Model" section in the main text), we write the original epidemic model as:

$$\begin{cases} S' = \mu \cdot (1 - K_1 - S) - \beta S \cdot (I + I_1), \\ S_1' = f_\rho \cdot \left(1 - \dfrac{S_1 + I_1 + R_1}{K_1}\right) - \beta S_1 \cdot (I + I_1) - \nu S_1, \\ I' = \beta S \cdot (I + I_1) - (\gamma + \mu) I, \\ I_1' = \beta S_1 \cdot (I + I_1) - (\gamma + \nu) I_1, \\ R' = \gamma I - \mu R, \\ R_1' = \gamma I_1 - \nu R_1. \end{cases} \tag{S5}$$

Here, $f_\rho = f(\rho) = \rho \lambda N_1$ represents the rate of incoming visitors. K_1 is the ratio of maximum capacity of visitors to the total population capacity. K_1 controls the upper bound of the magnitude of visitors in the model system (thus, generally, K_1 is fixed) S6.2. N_1 has the same meaning as in Table 1 and Eqn. (S3). N_1 is the ratio of total number of potential visitors (i.e., travelling-players) to the total population capacity (i.e., the sum of maximum visitors capacity and the size of local population, see Table 1, $(S + I + R + K_1)$ in model (S5)) For simplicity, we fix N_1 in this work. Model parameters are summarized in Table 1 in the main text.

Most visitors stay inside border (i.e., in the destination) for a considerably short period (three days, ν^{-1} in Table 1 and S6.3). Since $(S + I + R) + K_1 \equiv 1$ (i.e., the total population capacity, is scaled to unity) and $S_1 + I_1 + R_1 \leqslant K_1 < 1$, we have $(S + I + R) + (S_1 + I_1 + R_1) \leqslant 1$.

Under the quasi-steady-state assumption, which is widely adopted in within-host modelling studies [7, 5], we replace the term $\frac{S_1+I_1+R_1}{K_1}$ (in model (S5)) by $\frac{S_1+\left(1+\frac{\gamma}{\nu}\right)I_1}{K_1}$ (by forcing $R_1' = 0$) in order to eliminate equation of R_1. This approximation can be interpreted as that all R_1 come from I_1 and only $\frac{\gamma}{\gamma+\nu}$ of I_1 could transit to R_1 at any time (other part of I_1 simply leaving the system at rate ν). Thus, $R_1 \leqslant \frac{\gamma}{\gamma+\nu} I_1 \leqslant \frac{\gamma}{\nu} I_1$ (both γ and ν are positive), and then, $S_1 + I_1 + R_1 \leqslant S_1 + \left(1 + \frac{\gamma}{\nu}\right) I_1$. Since infected ($I_1$) visitors will quickly join R_1 class at the rate γ and the proportion of recovered visitors are relatively small, term $S_1 + I_1 + R_1$ is very close to $S_1 + \left(1 + \frac{\gamma}{\nu}\right) I_1$. Note that $\frac{\gamma}{\nu} I_1$ is simply the upper bound of R_1, and, after all, the effects of both I_1 and R_1 are little (compared with S_1) regarding to the visitors input.

After eliminating R' and R_1', we reformulate the epidemic model as,

$$\begin{cases} S' = \mu \cdot (1 - K_1 - S) - \beta S \cdot (I + I_1), \\ I' = \beta S \cdot (I + I_1) - (\gamma + \mu) I, \\ S_1' = f_\rho \cdot \left[1 - \dfrac{S_1 + \left(1 + \frac{\gamma}{\nu}\right) I_1}{K_1}\right] - \beta S_1 \cdot (I + I_1) - \nu S_1, \\ I_1' = \beta S_1 \cdot (I + I_1) - (\gamma + \nu) I_1. \end{cases}$$

This version is used in the main text.

For mathematical convenience, we fix $(S + I + R) + K_1 \equiv 1$ (i.e., the population threshold, or the total population capacity, is scaled to unity, 1). We also let $S_1 + (1 + \gamma/\nu)I_1 \leqslant K_1$, thus, $S_1 + I_1 + R_1 \leqslant K_1$ is guaranteed. Therefore, we have $(S + I + R) + (S_1 + I_1 + R_1) < 1$ in our complete model (see S3).

S4 Some Numerical Examples

The epidemics could be amplified by the uncontrolled visitor inflow, even when the basic reproduction number is low. Fig. S3(a) shows an epidemic becoming out of control with $\mathcal{R}_0$ declines (from 2.5 to 2.4) while the incoming visitor restriction fails (red line). The disease outbreaks can be controlled if the incoming visitors are restricted (i.e., by holding $\rho = 0.1$ unchange, see the green line). Since ρ^* is sensitive in a narrow range of $\mathcal{R}_0$ and r (see section "Results of Individual Equilibrium and Travelling Optimum" in main text), ρ^* could have very large change (e.g., from 0.1 to 0.99) with **slight** change on $\mathcal{R}_0$ (e.g., from 2.5 to 2.4 in Fig. S3(a)). The large variation in ρ^* could lead to the discrepancy between ρ^* and p^*. The decline of disease risk ($\mathcal{R}_0$) could avoid this discrepancy (by achieving $p^* = \rho^* = 1$). The increase of disease risk ($\mathcal{R}_0$) might also avoid this discrepancy (by achieving $p^* = \rho^* = 0$).

When the risk of disease (in term of $\mathcal{R}_0$) is higher than the perceived risk (i.e., the perceived risk is low), the local government is suggested to restrict visitor entrance. Otherwise, the actual proportion of the incoming visitor is likely to be greater than the optimal level (ρ^*). Fig. S3(b) shows the epidemic becoming out of control when $\mathcal{R}_0$ slightly rises and incoming visitors are not controlled (green line). The disease outbreak can be controlled by visitors entrance restriction (red and purple lines).

Fig. S3(c) shows the similar trend as the early stage of SARS epidemic (in Jan - Feb, 2003). The rapid increasing could be mainly due to the increased visitors during Chinese new year (see Fig. 2(a) of Ref. [10]). Namely the increase of visitors could lead to a disease outbreak.

S5 Sensitivity Analysis of Payoffs

Partial rank correlation coefficient (PRCC) analysis is deployed to assess the dependence of the model results on the parameters [7, 8, 9]. The ranges of model parameters used for the sensitivity analysis are summarized in Table 1 in the main text.

Fig. S4 shows the PRCCs between model parameters and individual payoff (E, see Eqn. (5)), and population risk level (Υ, Table 1 and S2.2) respectively. The ranges of model parameters are given in Table 1. Since "payoff" (the term in Fig. S4(a)) is the defined as the opposite number of "risk level" (the term in Fig. S4(b)), some model parameters have symmetric PRCC result with respect to level "0" (see the vertical grey dashed line in Fig. S4) on both panels. The PRCCs show that the results are most sensitive to the group of the relative risk (r), the basic reproduction number ($\mathcal{R}_0$), and the rate at which individuals leave the destination country (ν). Hence, these parameters should be the focus of data collection efforts during outbreaks when a travel policy must be decided. In Fig. S4(b), the basic reproduction number ($\mathcal{R}_0$) and relative risk (r) is

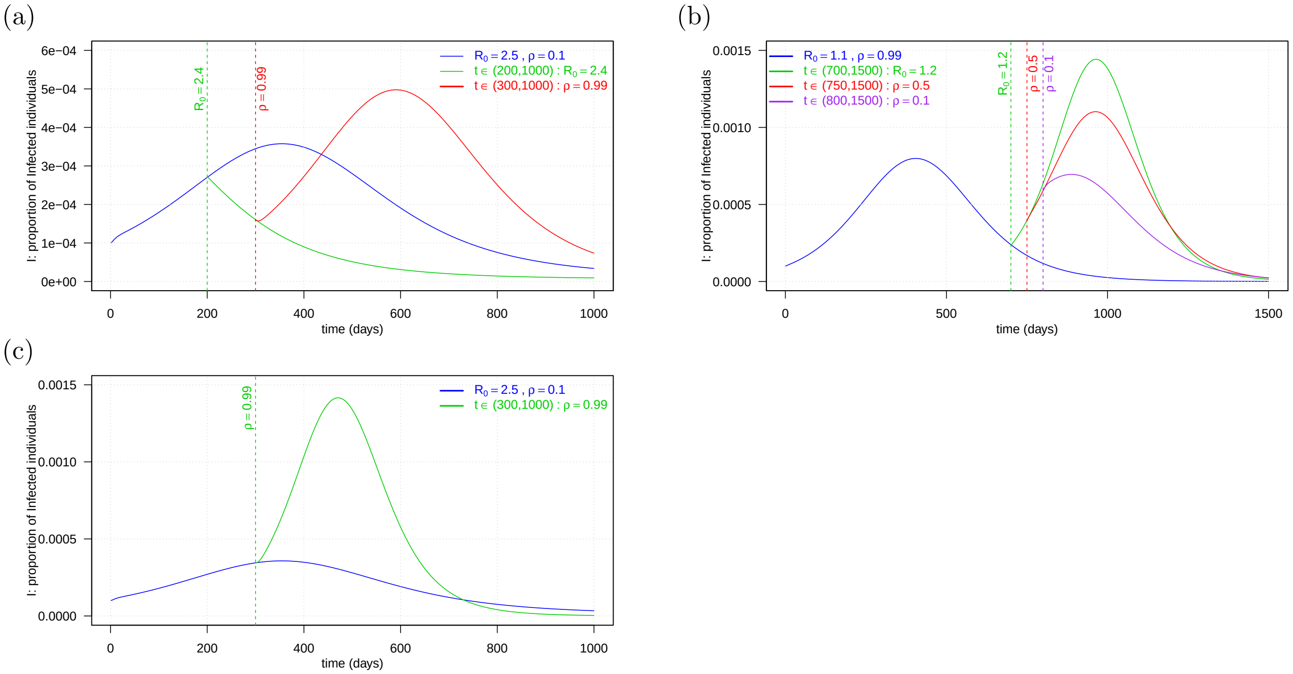

Figure S3: The simulation results of local infections (I) of epidemic model (panel (a) and (b), see Eqns. (S3)) and the SARS epidemic of China in 2002-03 (panel (c)). The baseline scenario contains that initial states are set as $[S(0), I(0), S_1(0), I_1(0)] = \left[\frac{1}{\mathcal{R}_0}, 1 \times 10^{-4}, \left(K_1 - 5 \times 10^{-6}\right), 5 \times 10^{-6}\right]$; with $\mathcal{R}_0 = 2.5$ and $\rho = 0.1$ for panel **(a)** and **(c)**, and $\mathcal{R}_0 = 1.1$ and $\rho = 0.99$ for panel **(b)**. Values of other parameters are on Table 1. In panel **(a)**, the blue line is the simulation results under baseline scenario of panel **(a)**; the green line is of basic reproduction number ($\mathcal{R}_0$) decreasing to 2.4 since the 201-st day (vertical green dashed line); based on the change of green line, the red line is of travelling proportion (ρ) increasing to 0.99 since the 301-st day (vertical red dashed line). In panel **(b)**, the blue line is the simulation results under baseline scenario of panel **(b)**; the green line is of basic reproduction number ($\mathcal{R}_0$) increasing to 1.2 since the 701-st day (vertical green dashed line); based on the change of green line, the red line is of travelling proportion (ρ) decreasing to 0.50 since the 751-st day (vertical red dashed line); based on the change of red line, the purple line is of travelling proportion (ρ) continually decreasing to 0.10 since the 801-st day (vertical purple dashed line). In panel **(c)**, the blue line is the simulation results under baseline scenario of panel **(c)**; the green line is of travelling proportion (ρ) increasing to 0.99 since the 301-st day (vertical green dashed line).

strongly positively related to the population risk level (Υ), and the visitors leaving rate (ν) is negatively related to Υ. Opposite results can be seen in Fig. S4(a) for the individual payoff.

S6 Interpretation and Value of Some Model Parameters

S6.1 Rate of visitors moving from outside status to inside status λ

The value of the mean period of a traveler stay outside border (λ^{-1}) can be estimated by referring to the "deadline" of cancellation of hotel room, flight or even car-rent for travelling usage. For example, according to cancellation policies of Airbnb (`https://www.airbnb.com/home/`

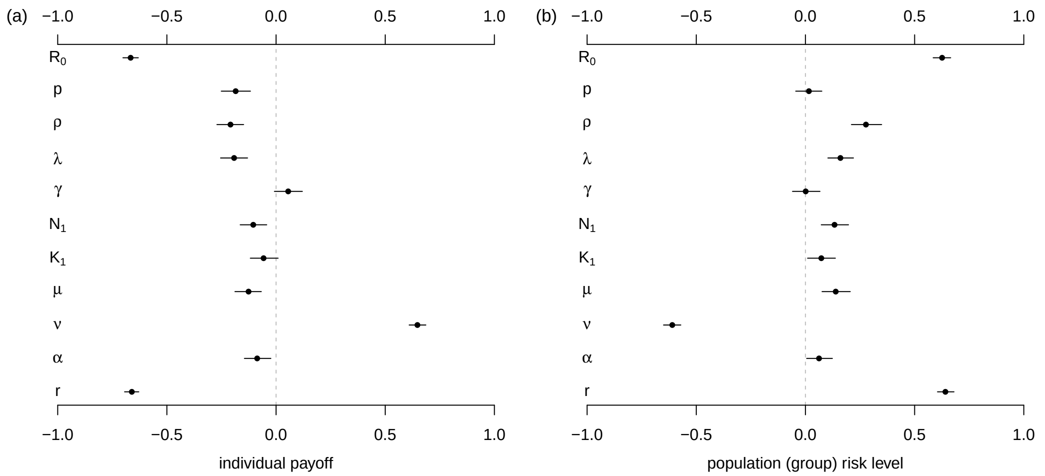

Figure S4: Sensitivity analysis results of (PRCCs) between model parameters and individual payoff (panel (a), see Eqn. (5)), and population risk level (Υ, in panel (b), see Table 1). The black dots are the estimated correlations and the bars represent 95% C.I.s. The ranges of model parameters are summarized on Table 1.

`cancellation_policies`), the waiver of refund charges can be considered for room-cancellation at least 1 day (with "flexible" policy) or 5 days (with "moderate" policy) in advance, thus we can make a rough estimation that $\lambda^{-1} \approx \frac{1}{2} \times (1+5) = 3$ days. According to Hong Kong Airline refund policies (`http://www.hongkongairlines.com/en_HK/flight/refund`), $\lambda^{-1} > 2$ days.

S6.2 Visitor capacity at destination K_1

According to the monthly travelling statistics (`http://partnernet.hktb.com/en/research_statistics/latest_statistics/index.html`) and travelling summary sheet (`http://partnernet.hktb.com/en/research_statistics/index.html`) from PartnerNet—Hong Kong tourism website for travel trade partners, there were approximate 58,000,000 travelers in Hong Kong of 2015 or 2016, and the local hotel room occupancy is roughly 87% over the whole period of time. Provided the information in S6.3, the local tavelling capacity of Hong Kong can be estimated as $\mathcal{N}K_1 = \frac{(58000000/87\%)\times 3}{365} \approx 550000$, here $\mathcal{N}$ denotes the number of total population capacity in Hong Kong (i.e., the sumation of upper bound of the number of travelers and local population, $\mathcal{N} = \mathcal{N}K_1 + \mathcal{N}\cdot(S+I+R)$). Given the popualtion statistics from World Bank (`https://data.worldbank.org/indicator/SP.POP.TOTL?locations=HK`), 7,300,000 is the number of local population in Hong Kong in 2015-16, thus $\mathcal{N} = \mathcal{N}K_1 + \mathcal{N}\cdot(S+I+R) = 550000 + 7300000 = 7850000$, and $K_1 = \frac{550000}{7850000} \approx 7.0\%$.

S6.3 Rate of visitors leaving destination ν

Referring to immigration department of the government of Hong Kong (`http://www.immd.gov.hk/eng/services/visas/visit-transit/visit-visa-entry-permit.html`), **Chinese** citizens can stay in Hong Kong for at maximal 7 days, and the majority of **non-Chinese** citizens can stay for roughly at maximal 15 days. According to the monthly travelling statistics from PartnerNet -

Hong Kong tourism website for travel trade partners (http://partnernet.hktb.com/en/research_statistics/latest_statistics/index.html), averagely, 75% of travelers are from mainland China and 25% are from other regions; for **Chinese** travelers, 50% of them are overnight passengers (expected to stay for $\frac{1}{2} \times (7+1) = 4$ days) and 50% them are one day visitors (expected to stay for $\frac{1}{2} \times (0+1) = 0.5$ day); for **non-Chinese** travelers, 66.67% of them are overnight passengers (expected to stay for $\frac{1}{2} \times (15+1) = 8$ days) and 33.33% them are one day visitors (expected to stay for $\frac{1}{2} \times (0+1) = 0.5$ day). Therefore, on average, one random-selected traveler would be expected to stay in Hong Kong for $\nu^{-1} = 75\% \times (50\% \times 4 + 50\% \times 0.5) + 25\% \times (\frac{2}{3} \times 8 + \frac{1}{3} \times 0.5) \approx 3$ days (thus, $\nu^{-1} = 3$ days).

S6.4 Relative risk r

The range of relative risk (r) can be approximated by simply checking the claim settlement odds of the travel insurance corresponding to the target place. For an example, according to travel insurance premium and coverage websites of Hang Seng Bank (https://bank.hangseng.com/1/2/personal/insurance/travel-leisure/travel-insurance/travel-premium and https://bank.hangseng.com/1/2/personal/insurance/travel-leisure/travel-insurance/travel-coverage), $r \approx 10^{-3}$.

S7 Further Discussion of Model Parameters

Relative risk $r = \frac{r_0}{r_1}$ (see Eqn. (S2) and Table 1) is the ratio of the "non-travelling" payoff ($E_0 = -r_0$, see main text) to the upper bound of the "travelling" payoff (i.e., $E_1 = -r_1$, see main text). The range of r could be obtain by referring to the claim-settlement-odds of the travel insurance with regard to the travelling destination (normally, $r \approx 10^{-3}$, see S6.4).

Number of visitors N_1 is the ratio of total number of potential visitors (i.e., game players) to the total population capacity. Provided total population capacity can be fixed in short term, the magnitude of N_1 is proportional to the number of potential visitors. We fix N_1 in this work. However, the number of potential visitors could be affected by seasonal factors (such as weather, school terms, holidays, etc.) and economic and politic factors (such as traffic expenditures, hotel fees, travelling policies [1], etc.), thus N_1 could be time-dependent in reality.

Agreement and conflict between ρ and p In Eqns. (S5) (and the epidemic model in the main text), $\frac{f_\rho}{\lambda} = \rho N_1$ is the proportion of visitors (outside border and about to be inside border shortly) to the total population capacity. ρ (see Table 1) is the proportion of potential visitors eventually becoming visitors correspond to the optimal travelling strategy selection. Therefore, we have $\rho = p^*$ (where p^* is individual's optimal travelling probability) under normal scenario (i.e., no serious disease outbreak, of which no restriction on travelling entry). However, during a serious disease outbreak, the local government will consider restricting travelling entry (in order to lower the number of visitors inside border) according to population's optimal travelling proportion (i.e., ρ^*), and this would change $\rho = \min\{p^*, \rho^*\}$. Numerical examples of local governmental

intervention on travelling entry (i.e., ρ) are discussed in section S4. Note that, under governmental intervention scenario, ρ should only equal to ρ^* if $\rho^* < p^*$ (otherwise $\rho^* \geqslant p^*$, $\rho = p^*$ is equivalent to normal scenario).

Period of visitors staying outside the border λ^{-1} is defined as the mean period for a visitor used to get inside the border (see Table 1). We stepwise the "visiting" population as in Path (1) in main text. The λ^{-1} is the mean period for a visitor evolving from a "visitor outside" border to a "visitor inside" border. Note that a "potential visitor" can only become a "visitor outside" if he has finished his final travelling decision (see S1). The knowledge of the range of λ^{-1} can be learnt by referring to the "deadline" of withdrawal of various travelling "services" (e.g., hotel, flight, etc., see S6.1). Therefore, the speed of health information spread could be related to λ^{-1} because that the updating of relevant information can "renew" individual's final decision (i.e., re-choose strategy). Therefore, higher speed of information spread is corresponding to lower value of λ^{-1}.

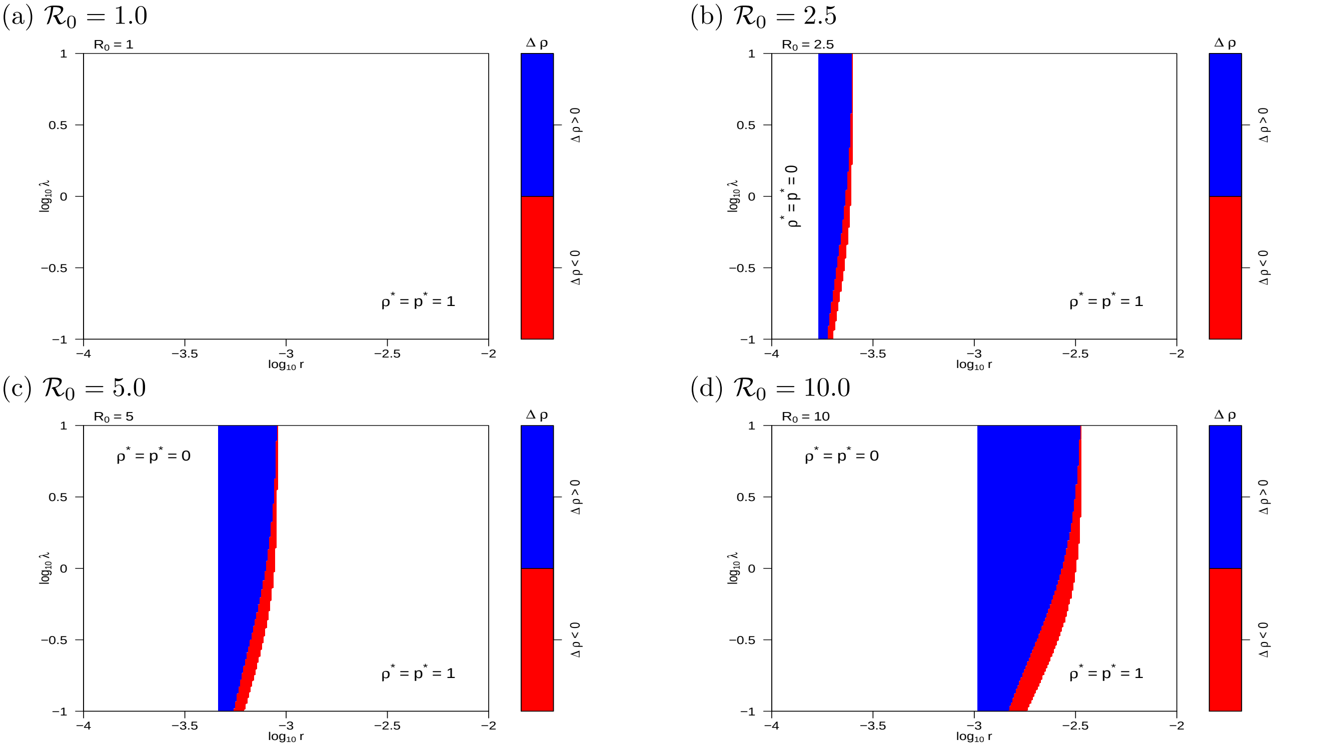

Figure S5: The relations among r, λ and $\Delta\rho$ (see main text) during epidemic (i.e., $\alpha = 1$) with $\mathcal{R}_0 = 1.0, 2.5, 5.0, 10.0$ for panel (a)-(d) respectively. The values of r and λ are in "$\log_{10}$" form. The color code of the difference of individual and population strategy, $\Delta\rho$, is shown on the color key. The white area (in each panel) represents $\Delta\rho = 0$ under two situations that $\rho^* = p^* = 0$ or 1. The values of other parameters are on Table 1.

Fig. S5 shows the relations among relative risk (r), rate of visitors pass border (λ) and $\Delta\rho$ during an epidemic. When λ increases, the discrepancy ($\Delta\rho$) of individual (p^*) and group optimum (ρ^*) appears under a wider range of relative risk (r). The discrepancy ($\Delta\rho$) shifts leftwards (the direction r increases) as $\mathcal{R}_0$ increasing. Particularly, p^* and ρ^* meet agreement (i.e., no discrepancy as $\rho^* = p^* = 1$) when $\mathcal{R}_0 = 1.0$ (which means disease cannot spread).

S8 Pre-existing immunity among visitors

For the model in the main text, we assume that all visitors are susceptible when entering the travel destination. In reality, this is not true. Pre-existing immunity of visitors could exist (e.g., the health authority of the visitors' home could recommend vaccination for visitors planning to go to a certain region where an epidemic is ongoing). The immunity level of the visitor population is dependent on a number of factors, including previous outbreaks, vaccination program policy and coverage, and the infection or vaccination history of the visitors, and may be estimated if the information is available. Thus, we denote

- P_{T} as the immunity level of the visitor population of a country;
- P_{O} as the immunity level of the rest of the population of the home countries of the visitors;
- P_{D} (i.e., term P in main text) as the immunity level of the local population of travel destination.

Then, the assumption $P_{\text{D}} = P_{\text{T}}$ or $P_{\text{D}} = P_{\text{O}}$, i.e., the immunity levels of local and origin populations are uniform, is unnecessary and can be relaxed. Moreover, the assumption $P_{\text{T}} = P_{\text{O}}$ can also be relaxed. In reality, $P_{\text{T}} > P_{\text{O}}$ could be common because (i) health authority of the visitors' home could recommend vaccination for visitors planning to go to an epidemic region; and (ii) vaccinated visitors are more likely to travel to an epidemic region.

After including P_{T}, the revised epidemic model becomes:

$$\begin{cases} S' = \mu \cdot (1 - K_1 - S) - \beta S \cdot (I + I_1), \\ S_1' = (1 - P_{\text{T}}) f_\rho \cdot \left[1 - \dfrac{S_1 + I_1 + R_1}{(1 - P_{\text{T}})K_1}\right] - \beta S_1 \cdot (I + I_1) - \nu S_1, \\ I' = \beta S \cdot (I + I_1) - (\gamma + \mu) I, \\ I_1' = \beta S_1 \cdot (I + I_1) - (\gamma + \nu) I_1, \\ R' = \gamma I - \mu R, \\ R_1' = \gamma I_1 - \nu R_1, \end{cases}$$

with all the terms remaining unchanged, except for inclusion of $(1-P_{\text{T}})$ in $(1-P_{\text{T}})f_\rho \cdot \left[1 - \frac{S_1+I_1+R_1}{(1-P_{\text{T}})K_1}\right]$.

We note that we could include one more equation,

$$X_1' = P_{\text{T}} f_\rho \cdot \left[1 - \frac{S_1 + I_1 + R_1 + X_1}{(1 - P_{\text{T}})K_1}\right] - \nu X_1,$$

where the additional state X_1 denotes visitors being protected against the disease, and the term $\frac{S_1+I_1+R_1}{(1-P_{\text{T}})K_1}$ (in the revised model) should originally be written as $\frac{S_1+I_1+R_1+X_1}{K_1}$ (the same as in Eqn. X_1'). Since the magnitudes of both I_1 and R_1 are relatively small with respect to S_1 and X_1, we ignore the effects of I_1 and R_1 on the incoming visitors rate. Thus we have

$$S_1' \approx (1 - P_{\text{T}}) f_\rho \cdot \left[1 - \frac{S_1 + I_1 + R_1 + X_1}{K_1}\right] - \nu S_1.$$

We can easily see that P_{T} of f_ρ joins in X_1, $(1-P_{\mathrm{T}})$ of f_ρ joins in S_1, and the leaving rates of X_1 and S_1 are the same as ν. To eliminate term X_1, we have $X_1 \approx \frac{P_T S_1}{(1-P_T)}$; therefore,

$$\frac{S_1+I_1+R_1+X_1}{K_1} \approx \frac{S_1+I_1+R_1}{(1-P_T)K_1},$$

as shown in the above revised model.

The term $(1-P_{\mathrm{T}})$ can be interpreted to mean that the protected visitors (P_{T}) are directly removed from the system (not by joining R_1, but by being "completely" removed from the model system), and the effect on the visitor input rate is partly reflected by "reducing" the local visitor capacity (i.e., replacing K_1 by $(1-P_{\mathrm{T}})K_1$). In this work, P_{T} is fixed to 0. Then, a new simplified model can be derived (from the revised model) by following the same method in S3 (by eliminating R and R_1). Since we regard P_{T} as a fixed nonzero constant (i.e., $P_T \neq 0$) during a short time period, and mathematically speaking, the effect of P_{T} can be transformed into a reduction of the magnitudes of f_ρ and K_1 [11], the main results in this work will hold for the revised epidemic model.

S9 Risk of visitors bringing the disease back to their home country

For the analysis in main text, for simplicity, we assume that visitors do not bring diseases back to their home country. This assumption is clearly overly optimistic. To amend this shortcoming, we may introduce one additional probabilistic factor of the risk level and obtain an improved travelling risk function

$$\Upsilon = \Upsilon(\rho,\pi) = N_1 \cdot \left[\rho \cdot \alpha\phi(\rho) \cdot (1+\pi \cdot \frac{\varrho}{r_1}) \cdot r_1 + (1-\rho)r_0\right],$$

where π is the average probability that the disease is brought back to the home country of a traveller, and ϱ is the average payoff of the disease spreading in a randomly selected home country.

Generally, we note that $\varrho > r_1$, since the consequences of a disease spreading in a region are presumed to be more serious than the consequences of a single individual being infected from a utilitarian point of view. We fix the ratio of $\frac{\varrho}{r_1}$ and use a similar idea as $r = \frac{r_0}{r_1}$. We view $(1+\pi \cdot \frac{\varrho}{r_1})$ as a scaler and assign a value to π. Thus the results of our original framework still hold, namely, the epidemic risk level of the travelling population, as listed in Table 1 in main text is a simplified version when $\pi = 0$.

References

[1] Cui JA, Takeuchi Y, Saito Y. Spreading disease with transport-related infection. Journal of theoretical biology. 2006;239(3):376-90.

[2] Bauch CT, Galvani AP, Earn DJ. Group interest versus self-interest in smallpox vaccination policy. Proceedings of the National Academy of Sciences of the United States of America. 2003;100(18):10564-7.

[3] Bauch CT, Earn DJ. Vaccination and the theory of games. Proceedings of the National Academy of Sciences of the United States of America. 2004;101(36):13391-4.

[4] Ciupe SM, Heffernan JM. In-host modeling. Infectious Disease Modelling. 2017.

[5] Ben-Shachar R, Koelle K. Minimal within-host dengue models highlight the specific roles of the immune response in primary and secondary dengue infections. Journal of the Royal Society Interface. 2015;12(103):20140886.

[6] Molina C, Earn DJD. Game theory of pre-emptive vaccination before bioterrorism or accidental release of smallpox. Journal of The Royal Society Interface. 2015;12(107):20141387.

[7] Ciupe SM, Heffernan JM. In-host modeling. Infectious Disease Modelling. 2017.

[8] Wu J, Dhingra R, Gambhir M, Remais JV. Sensitivity analysis of infectious disease models: methods, advances and their application. Journal of The Royal Society Interface. 2013;10(86):20121018.

[9] Gao D, Lou Y, He D, Porco TC, Kuang Y, Chowell G, Ruan S. Prevention and control of Zika as a mosquito-borne and sexually transmitted disease: a mathematical modeling analysis. Scientific reports. 2016;6:28070.

[10] Feng D, De Vlas SJ, Fang LQ, Han XN, Zhao WJ, Sheng S, Yang H, Jia ZW, Richardus JH, Cao WC. The SARS epidemic in mainland China: bringing together all epidemiological data. Tropical Medicine & International Health. 2009;14(s1):4-13.

[11] Earn DJ, Rohani P, Bolker BM, Grenfell BT. A simple model for complex dynamical transitions in epidemics. Science. 2000;287(5453):667-70.
